# Recognition of Unknown Conserved Alternatively Spliced Exons

**DOI:** 10.1371/journal.pcbi.0010015

**Published:** 2005-07-08

**Authors:** Uwe Ohler, Noam Shomron, Christopher B Burge

**Affiliations:** Department of Biology, Massachusetts Institute of Technology, Cambridge, Massachusetts, United States of America; Fred Hutchinson Cancer Research Center, United States of America

## Abstract

The split structure of most mammalian protein-coding genes allows for the potential to produce multiple different mRNA and protein isoforms from a single gene locus through the process of alternative splicing (AS). We propose a computational approach called UNCOVER based on a pair hidden Markov model to discover conserved coding exonic sequences subject to AS that have so far gone undetected. Applying UNCOVER to orthologous introns of known human and mouse genes predicts skipped exons or retained introns present in both species, while discriminating them from conserved noncoding sequences. The accuracy of the model is evaluated on a curated set of genes with known conserved AS events. The prediction of skipped exons in the ~1% of the human genome represented by the ENCODE regions leads to more than 50 new exon candidates. Five novel predicted AS exons were validated by RT-PCR and sequencing analysis of 15 introns with strong UNCOVER predictions and lacking EST evidence. These results imply that a considerable number of conserved exonic sequences and associated isoforms are still completely missing from the current annotation of known genes. UNCOVER also identifies a small number of candidates for conserved intron retention.

## Synopsis

Alternative splicing is a process in which more than one protein variant can be produced from one gene: Specific parts of the mRNA precursor are included or excluded during the processing into the mature transcript. It is very prevalent in mammalian genomes, and variants are often specific for particular cell types, developmental states, or environmental changes. The identification of such variants has until recently relied solely on the sequencing and comparison of expressed sequence tags (ESTs), but the number of available ESTs is not large enough to cover all variants under all conditions.

Ohler et al. have now devised a comparative genomics algorithm based on a pair hidden Markov model, which identifies parts of genes that are alternatively spliced and have not been observed in ESTs. Starting from known annotated genes conserved in human and mouse, they scan corresponding intron pairs of these genes to identify conserved sequences that match the model. Experimental validation of a number of new predictions show that the approach can successfully uncover splice variants that are as yet unknown and not part of the large libraries of ESTs. Together with recently proposed complementary computational methods, this approach helps us to complete our knowledge about the transcript diversity created by alternative splicing.

## Introduction

Almost all protein-coding genes of humans and other mammals have a split structure with several exons and introns. Intronic sequences are removed from the primary transcript by the process of pre-mRNA splicing [1], an essential step in eukaryotic gene expression. The number of functional variants generated from one transcript can be greatly increased by alternative splicing (AS), in which one or more exons or parts thereof are skipped, or an intron is retained, when compared to a different transcript from the same gene [2–4]. By this mechanism, an organism can generate several protein isoforms from a single gene, potentially leading to huge numbers of protein variants, and AS is an important means of gene regulation, being frequently used during development or in differentiation. It is also a very common event: even conservative estimates put the fraction of human genes with more than one isoform at 40% [5], with similar rates estimated in all animals [6]. The basic types of AS are exon skipping, intron retention, and alternative 5′ and 3′ splice site usage, with exon skipping being the most prevalent in mammals [7–10]. To date, AS events have been identified on a large scale primarily from comparisons and alignments of expressed sequence tag (EST) and cDNA sequences, and databases based on these alignments have been described [8,10,11]. Ab initio prediction of AS events from one genomic sequence alone has been attempted only rarely: Computational screening of introns for sequences similar to neighboring exons revealed candidate duplicated exons, which may be involved in mutually exclusive splicing [12]. A hidden Markov model (HMM) sampling approach can detect likely variants of complete gene structures [13]. These studies, however, did not experimentally verify predicted AS events.

Despite the large number of ESTs that have been sequenced from a variety of organisms and tissues, the coverage of the transcriptome still remains limited, especially for genes expressed at lower levels or under limited conditions. It becomes increasingly hard to distinguish functional but rare EST-detected variants from nonfunctional isoforms and artifactual sequences contained in the libraries [7]. At least in mammals, the less common isoforms are often not conserved, in contrast to the high degree of conservation seen for the more common variants [14,15]. Exons subject to nonconserved skipping events are significantly different from alternative conserved exons (ACEs, pairs of orthologous human/mouse exons both subject to exon skipping), being less likely to preserve reading frame and more likely to contain in-frame stop codons, suggesting that a significant fraction does not lead to functional proteins [3]. ACEs also tend to be flanked by long, highly conserved intronic sequences, possibly because of the presence of sequence elements required to regulate inclusion of the exons in specific cell types or conditions. Regions containing ACEs are thus often among the most highly conserved segments in the human genome [16,17]. Two non-EST-based computational approaches have made use of these specific features to successfully classify conserved exons as to whether they are subject to skipping or not [17,18], which confirmed that current coverage of splicing isoforms by ESTs alone is still limited, but that most EST-derived skipping events may in fact not be conserved. Regarding intron retention events (IREs), a recent study estimated that they occur in about 15% of human genes [19]; however, stricter requirements lower this estimate to 5% [7,19], and half of these cases are within the untranslated region and thus do not alter the encoded protein. Only ten of the reliably determined IREs, coding or noncoding, were found to be clearly conserved between human and mouse, based on currently available EST and cDNA evidence, suggesting that this mode of regulation is not common in mammalian genes.

## Results

### Design of a Pair HMM to Discover Conserved AS

Given that conserved coding AS events have the potential to alter protein isoforms under tightly regulated circumstances, these sequences should be among the functionally most important segments of the genome. Computational approaches to predict ACEs [17,18] demonstrated that inferring AS events from ESTs alone will miss a considerable fraction of conserved skipping events: those for which current EST libraries contain only those isoforms that include the exon. Presumably, this is caused by the fact that the majority of isoforms include the ACE under consideration. Here, we set out to develop a complementary approach to predict those conserved AS events in which the majority of isoforms do not include the ACE or retained intron, and for which the exonic sequence subject to AS is thus completely absent in available EST sequences and has not been described before.

To systematically identify such previously unknown ACEs and IREs missed by comparative gene-finding and cDNA and EST alignments, we developed a system for comparative prediction of mammalian conserved coding AS events termed UNCOVER (for “unknown conserved variable exon recognition”). UNCOVER is based on a pair HMM (pHMM) [20–22], a probabilistic model that can be used to obtain an optimal alignment and simultaneous annotation of two sequences. A pHMM consists of states that can be either pair states, which contain a probability distribution on the occurrence of pairs of aligned nucleotides, or single states, which model nucleotides in one sequence but not the other, thus describing insertions and deletions. Different states are used to model different patterns of conservation, e.g., the distribution in a state for the third codon position will typically contain higher probabilities for mismatches than the one for the first or second position, and those mismatches not changing the encoded amino acid will be more frequent than others. While computing the optimal alignment, a pHMM labels the alignment with the states that were used in the process, and the aligned sequence can be parsed into functional categories based on the labels.

**Figure 1:**
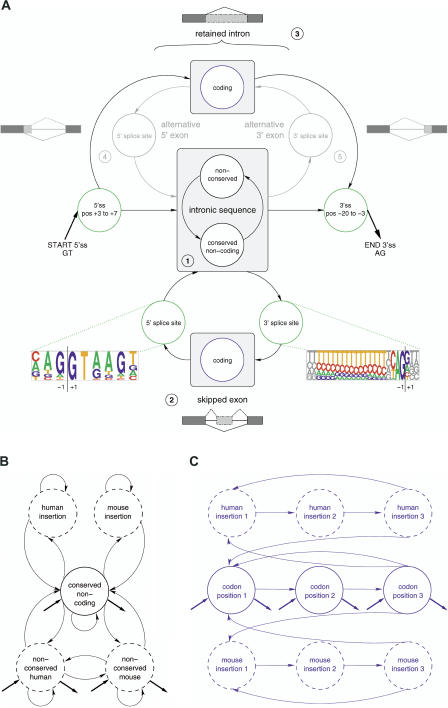
Structure of the UNCOVER pHMM The model is used to globally align a pair of orthologous human/mouse introns and detect conserved coding AS events. (A) A schematic overview of the model architecture, with circles indicating groups of functionally related states. For accurate splicing, the two ends of an intron must be precisely determined by the splicing machinery. The prominent sites for this process are the 5′ splice site (5′ss) at the junction between the upstream exon and the intron and the 3′ splice site (3′ss) at the junction of the intron and the downstream exon. As reference, pictograms of the mammalian 5′ splice site and 3′ splice site are depicted, in which the letters at individual positions are scaled according to their frequency. We restrict ourselves to U2-type splice sites with perfectly conserved GT–AG dinucleotides. The alignment always starts with the conserved 5′ splice site after the initial GT dinucleotide. The transitions of the model then allow it to pursue several paths, corresponding to different types of AS, indicated by small icons. (1) The “default” is to observe conserved or nonconserved noncoding sequence, possibly alternating between these two. (2) Transitions to an ACE sequence of conserved {3′ splice site, skipped exon, 5′ splice site} are possible at any time, and can also occur more than once. (3) An IRE is modeled by going from a 5′ splice site to a 3′ splice site by only passing through a coding submodel. (4) and (5) An early exit from this codon model through another 5′ splice site leads to an alternative 5′ exon at the beginning of the sequence, or correspondingly to an alternative 3′ exon at the end. The alignment is fixed on the right side by the 3′ splice site at the end of the intron. All splice site states are first-order pair states not allowing for insertions or deletions. The 5′ splice site part of the model covers 9 nt (3 nt in the exon, plus the conserved GT and the following 4 nt in the intron); the 3′ splice site is 23 nt long (18 nt and the conserved AG in the intron plus 3 nt in the exon). (B and C) A detailed view of the noncoding intronic submodel (B) and a close-up of the coding submodel (C), with closed circles representing pair states and dashed circles representing single states. Thick straight arrows indicate the allowed start and end states of the submodels. The noncoding conservation (B) is modeled by a first-order pair state, allowing insertions and deletions of individual nucleotides. The null model contains single first-order states representing nonconserved human and mouse intronic sequences. The coding states (C) comprise three second-order pair states for nucleotides in the three codon positions, as well as three second-order single states each for human and mouse to capture species-specific codon insertion/deletion events. The transition matrix ensures that only those insertion/deletion events covering complete codons are admissible.

**Figure 2:**
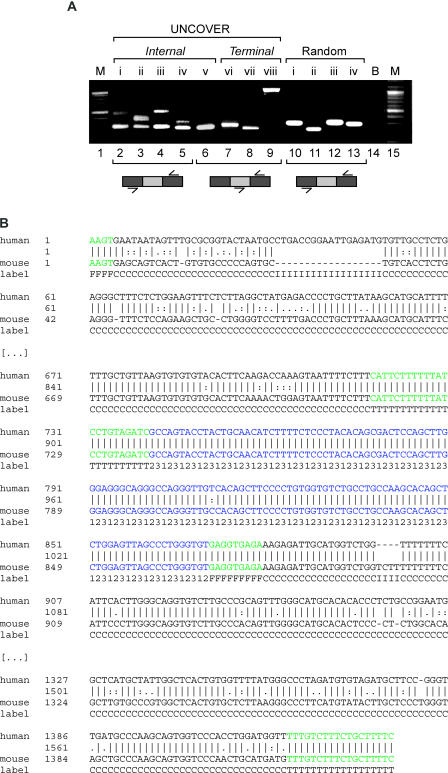
Experimental Validation of UNCOVER Predictions (A) RT-PCR validation of newly identified alternative exons with no prior EST evidence. Lane numbers are given in Arabic numerals below the gel; sample numbers of new verifications and negative controls are in Roman numerals above. Lanes 2–5 were verified using flanking primers and therefore show two bands each, the larger one corresponding to the event including the newly identified ACE. Lanes 6–9 used a primer internal to the newly identified exon and therefore only show one band each. Lanes 10–13 are typical examples of ten randomly selected introns in the ENCODE target regions that were not predicted to harbor AS events. Lane 14 shows a blank reaction control without adding template. Lanes 1 and 15 contain size markers spaced at 100 nt intervals, with the strong bands corresponding to 1,000 and 500 nt. Ensembl ID pairs for the known exon upstream of the validated new one and the corresponding gene are as follows: internal exons, lanes 2–6: ENSE00000881911.1:ENSG00000004866.5, ENSE00000862512.1:ENSG00000126217.3, ENSE00001201432.1:ENSG00000168781.5, ENSE00001146476.1:ENSG00000168781.5, and ENSE00001084095.4:ENSG00000164402.2; terminal exons, lanes 7–9: ENSE00001379673.1:ENSG00000159140.5, ENSE00001046164.1:ENSG00000067369.1, and ENSE00000952769.2:ENSG00000142183.3 (a known case as positive control); random negative controls, lanes 10–13: ENSE00001321652.4:ENSG00000161980.2, ENSE00000868377.2:ENSG00000102125.4, ENSE00001239587.1:ENSG00000100220.2, and ENSE00001307891.1:ENSG00000185721.1. (B) Example UNCOVER alignment of a newly detected ACE. Aligned nucleotides are connected with a vertical dash in case of identity, a colon in case of a transition, and a dot in case of a transversion. The alignment is labeled with the types of the states that lead to the most likely alignment: C, conserved noncoding sequence; F, 5′ splice site; I, nonconserved intronic sequence; T, 3′ splice site; 1, 2, and 3, coding sequence, with the number giving the position in a codon. The detected ACE is flanked by highly conserved noncoding sequence, a characteristic of true ACEs. The sequence shown corresponds to the event in sample i in (A).

The UNCOVER pHMM was designed to specifically align one orthologous human/mouse intron pair at a time and to predict whether it potentially harbors undiscovered AS events (Figure 1). The model states describe the probability of aligned nucleotide pairs in the 3′ and 5′ splice sites, coding regions, and noncoding alignable regions, as well as single nucleotides in nonalignable regions (i.e., insertions or deletions in the human sequence when compared to mouse). The transition probabilities of the model connect the states in different ways corresponding to submodels for none or any one of four basic AS events: skipping, retention, and alternative 5′ and 3′ exons. A labeled UNCOVER alignment can thus predict whether a conservation pattern seen in the intron pair fits better to conserved noncoding sequence, to coding sequence throughout—suggesting intron retention—or to the conserved sequence of {3′ splice site, coding exon, 5′ splice site} somewhere within the intron pair, suggesting the presence of an ACE (see Figure 1 for a detailed description of the model, and Figure 2 for an example alignment). The UNCOVER submodels for alternative 5′ and 3′ exons are at this point used only to achieve better discrimination between the different AS types, and are not analyzed in detail in this study.

Candidate structures scored by current ab initio gene-finding algorithms are limited in that they have to fulfill the restrictions of the whole gene model—including presence of an open reading frame throughout and distributions on the expected length of exons. The UNCOVER model as shown does not impose these restrictions and thus has the potential to detect conserved events missed by computational gene finders. It can be used to predict new AS events in two species simultaneously, or to provide additional evidence for a conserved AS event in case of limited EST coverage or ESTs from only one of two species. An advantage of our pHMM is that, in addition to identifying AS events, it also identifies conserved noncoding sequences, potentially containing *cis*-regulatory elements for splicing or transcription. UNCOVER per se identifies any kind of coding sequence fitting the pHMM model, which means that predicted skipped exons may in fact simply be exons missed by the existing annotation that are conserved but not alternatively spliced. In practice, however, the pipeline to determine the input intronic regions uses annotations of conserved gene structures, which are generally inferred from EST and cDNA evidence, and by definition most true positive predicted exons are thus skipped exons.

### Application of UNCOVER on a Curated Dataset of Known Skipped Exons

To establish a baseline for how well ACEs can be detected with our approach, we collected 241 orthologous introns containing known ACEs derived from human and mouse EST and cDNA alignments, ranging in length from about 250 nt to about 93,000 nt. UNCOVER made a total of 309 predictions with 210 true positives (Table 1), successfully pinpointing the exact location of the ACEs: 89% of true positive UNCOVER predictions identified at least one splice site exactly. The inexactness of the remaining 11% reflected the strong sequence conservation around ACEs, which makes it difficult to infer the exact location of the correct splice sites in some cases. For comparison, we performed a simple BLASTN [23] analysis, keeping all hits longer than 30 nt with *E* values smaller than 10^−10^. This resulted in 667 predictions, out of which 253 overlapped with 233 known exons. However, not a single hit corresponded to the exact exon boundaries. BLASTN can thus detect the rough locations of the great majority of ACEs in this set, but in an extremely unspecific manner; using TBLASTX instead of BLASTN gave highly similar results. Retaining only the best hit with at least 70% sequence identity but independent of *E* value resulted in 212 hits (88%) overlapping ACEs. The UNCOVER detection rate is thus virtually identical to the best BLAST hit analysis, but without making any unrealistic assumptions as to whether or how many ACEs may be present in an intron (and, importantly, UNCOVER predictions usually have one or both splice sites correct).

**Table 1:**
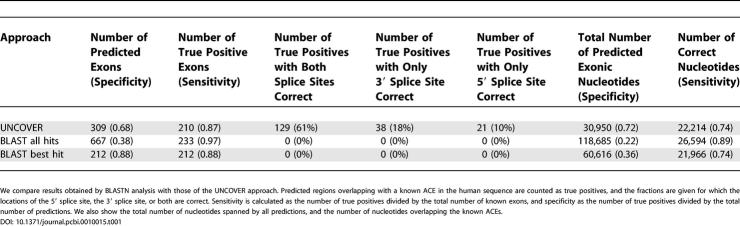
Prediction Results on a Known Set of 241 Conserved Skipped Exons

We compare results obtained by BLASTN analysis with those of the UNCOVER approach. Predicted regions overlapping with a known ACE in the human sequence are counted as true positives, and the fractions are given for which the locations of the 5′ splice site, the 3′ splice site, or both are correct. Sensitivity is calculated as the number of true positives divided by the total number of known exons, and specificity as the number of true positives divided by the total number of predictions. We also show the total number of nucleotides spanned by all predictions, and the number of nucleotides overlapping the known ACEs.

As an alternative to probabilistic sequence models, the Ka/Ks test has recently been applied to the problem of comparative gene finding. This is an established method to detect adaptive molecular evolution, based on the observation that coding sequences are generally under selection to conserve amino acid sequence. In an application of the Ka/Ks test to gene finding [24], 92% of internal exons passed the test at a *p-*value of 0.05. However, only 47% of the tested conserved skipped exons in our set of 241 exons passed the test at the same *p-*value, even under the assumption of knowing the exact exon boundaries. This is apparently due to the smaller size of the skipped exons (median 84 nt compared to 123 nt in the set of constitutive exons used in [24]) and the higher rate of synonymous sequence conservation of ACEs compared to constitutive exons (see Protocol S1 for details). The Ka/Ks test therefore has inherent limitations when applied to detect alternatively spliced exons.

### Analysis of the ENCODE Target Regions

As an application of UNCOVER on a genomic scale, we focused on the 1% subset of the human genome known as the ENCODE (Encyclopedia of DNA Elements) target regions, currently the subject of comprehensive experimental and computational analyses [25]. UNCOVER made 135 predictions in 73 out of a total of 1,776 orthologous introns (4.1%), located in 40 out of 323 genes (12.4%). In comparison, there were 982 BLAST hits to 321 introns with the thresholds set as above, more than seven times as many hits at a similar level of sensitivity. A total of 42 UNCOVER predictions corresponded to either annotated human skipped exons or sequences covered by human ESTs in dbEST (as of August 23, 2004): 15 matched annotated ACEs in known Ensembl genes; seven matched annotated Ensembl EST genes or VEGA (the manually curated Vertebrate Genome Annotation database [26]; http://vega.sanger.ac.uk) genes; and three matched spliced ESTs not corresponding to any annotation, indicating the presence of yet unannotated ACEs in the genes *LUC7L, C16orf35,* and *CDH2*. The remaining predictions matched unspliced ESTs corresponding to 11 intronic regions. Many of these ESTs were polyadenylated, and one of the matches was annotated as an alternative terminal exon of an EST gene. Indeed, we observed that with only one exception, these UNCOVER predictions were located in the 3′ terminal region of the genes. The location of these putative terminal exons cannot be expected to be exactly predicted by UNCOVER, as they do not end with a 5′ splice site and contain 3′ untranslated sequence.

For experimental validation, we selected those 20 introns containing predicted ACEs without any EST evidence that were flanked on both sides by strong splice sites. We followed an RT-PCR sequencing protocol in a set of eight adult human tissues and HeLa cells, and confirmed expression of the flanking exons for 15 out of the 20 tested introns (i.e., in five cases, we could not observe any expression in the selected tissues using multiple sets of primers). For five out of these 15, we repeatedly observed two PCR bands, with the sequence of the smaller product matching the exons flanking the predictions, and in one additional case, we saw expression of a product using primers placed inside the predicted ACE and a neighboring exon. In three of these six cases (including the gene *ST7*), the sequence of the alternative product included the UNCOVER predicted exons, showing that our approach led to the discovery of new ACEs expressed at low levels that had not yet been covered despite the availability of more than 5 million human ESTs (Figure 2; see also Dataset S1 for details). One case *(CRAT)* corresponded to a skipped exon in which only a small part in the middle was conserved between human and mouse, and which could therefore not be predicted by UNCOVER. In the remaining two cases (including *MCF2L*), the included alternative sequence did not match any sequence in the nonredundant GenBank database, suggesting gaps or misassemblies in these introns. Furthermore, we confirmed two of the ten potential new alternative terminal exons, using primers placed inside the predicted exon and the immediately upstream exon. Not counting the cases with nonmatching sequence, we therefore report here eight conserved AS events—five verified by RT-PCR based on de novo predictions plus three with spliced EST evidence—in addition to 15 known events present in the Ensembl annotation of the ENCODE regions (as of August 2004), and provide additional support for eight more ACEs that have only been annotated as part of Ensembl EST genes or cross-species homology.

### A Genome-Wide Search for Conserved Retained Introns

Turning to conserved intron retention, we extended the UNCOVER analysis to the whole genome. Our analysis spanned a total of 84,233 orthologous intron pairs, 46 times the number within the ENCODE region, which covers 1% of the nucleotides in the genome but is somewhat gene rich. Despite this large number of introns, and without assumptions on the reading frame of the upstream exon, only 23 were predicted to be more likely to be conserved IREs with coding potential than to harbor conserved noncoding sequence (see Table 2 and Dataset S2). Out of these, 12 were covered by human ESTs (as of October 25, 2004), with a total of ten annotated as known or EST genes. The length of 12 candidates was a multiple of three, and 13 out of 19 for which we could determine the open reading frame from full-length cDNAs were predicted to continue in the frame of the upstream exon. Given evidence of length, reading frame, EST coverage, and the presence of protein domains spanning the candidate IRE, another four (among them *PAX6* and *PCDH17*) in addition to the ten already annotated can be considered highly likely IREs, and an additional two involve splicing of an alternative 5′ splice site in a mutually exclusive fashion to one of the neighboring exons.

**Table 2:**
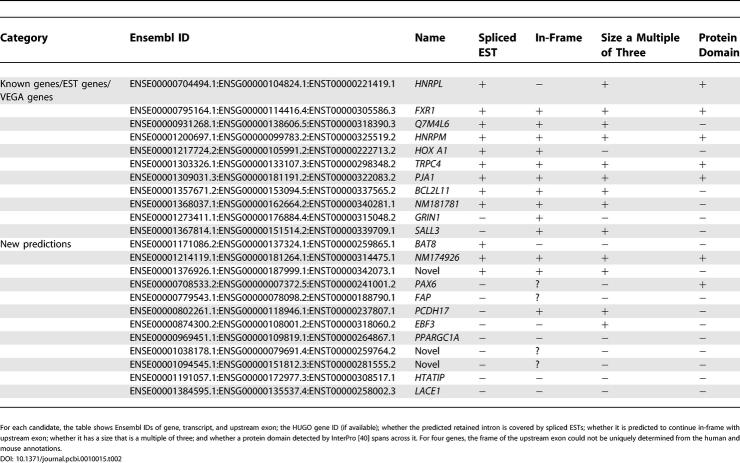
Predicted Conserved Coding IREs and Their Evidence

For each candidate, the table shows Ensembl IDs of gene, transcript, and upstream exon; the HUGO gene ID (if available); whether the predicted retained intron is covered by spliced ESTs; whether it is predicted to continue in-frame with upstream exon; whether it has a size that is a multiple of three; and whether a protein domain detected by InterPro [40] spans across it. For four genes, the frame of the upstream exon could not be uniquely determined from the human and mouse annotations.

## Discussion

We propose a comparative sequence analysis approach to detect hitherto unknown and alternatively spliced conserved exons, followed by experimental validation. Considering the 53 introns with UNCOVER predictions in the ENCODE region that do not contain annotated skipped exons, and adjusting the number by the sensitivity (87%) and specificity (68%) of UNCOVER on the curated ACE dataset, leads to an estimated total of 53(0.68)/(0.87) = 41 ENCODE introns containing ACEs not currently annotated. This shows that even for known and well-studied genes, current EST coverage is far from providing a complete picture of AS. Scaled up to the whole genome, which contains 46 times the number of introns in the ENCODE region, about 1,900 introns may harbor as yet unknown ACEs. Since specificity may be overestimated somewhat using the curated ACE dataset, as a lower estimate, a straight extrapolation of the so far experimentally verified ACEs suggests that at least several hundred ACEs are currently still awaiting discovery. We expect that UNCOVER will therefore be especially useful when turning to regions of the genome less covered by ESTs [27] than the ENCODE targets. On the other hand, retention of translatable introns does not appear to play a major role in generating conserved protein-coding isoforms in mammals. We do not rule out a common role for nonconserved regulated intron retention or conserved IREs in UTRs or in species other than mammals.

Considering the RT-PCR results, the isoforms that include the newly verified exons are expressed at lower levels than the isoforms in which the exons are skipped. This is in accordance with the lack of support in EST data: were the isoform with the exon included the major one, we would expect it to have been observed in the EST data. Detailed measurements of the frequency of individual AS events, such as for the well-studied cell-surface receptor *CD44*, showed that the inclusion of functional alternatively spliced exons can indeed be much less common than skipping [28]. A number of points argue for the functional relevance of our newly detected minor isoforms: we are usually able to amplify them by placing primers in the flanking exons (see Figure 2); they are expressed in a tissue-specific manner in human, and we observe expression in mouse as well (Figure S1); and their sequence is conserved not only in mouse but in a number of other vertebrate species (see Dataset S1).

In its current state, UNCOVER is designed to predict only fully coding exons. In addition to an easy adaptation to pairs of non-mammalian species such as nematodes or insects, further development of UNCOVER could lead to removal of this restriction to include exons with in-frame stop codons and noncoding 3′ ends. This should enable us to better predict terminal exons that are only partly coding: we showed that these can be predicted by the current version of UNCOVER, but an explicit model of noncoding conservation and polyadenylation sites would undoubtedly lead to improvements. Furthermore, including in-frame stop codons may allow predictions of additional ACEs subject to nonsense-mediated decay (NMD), a mechanism that degrades transcripts containing premature termination codons [29]. NMD has been proposed as an important mechanism for gene regulation in conjunction with AS [9]. A PCR verification of predictions subject to NMD could be done following knockdown of the important enzymes in the NMD pathway, to be able to accumulate and amplify the transcripts. To gain additional confidence in such predictions, UNCOVER ought to be extended to more than two species, which should additionally allow reliable prediction of alternative 5′ and 3′ splicing that may lead to isoforms differing by only a few nucleotides. This can be done in a way similar to an approach based on probabilistic phylogenetic models [30].

Recent independent methods based on comparative genome analysis [17,18,31], which can be regarded as complementary to the work described here, have been successful in classifying known conserved exons as skipped or constitutive. These approaches are based on methodology from statistical learning theory, and a true integration with a probabilistic approach such as UNCOVER is not straightforward. However, they could be easily used to filter our predictions. A genome-wide verification of such predictions is planned, which should contribute to completing our picture of the extent and prevalence of conserved AS.

## Materials and Methods

### Training and test datasets.

A comprehensive set of reliably annotated exon–intron structures of mammalian genes, including information about alternative structures as well as conservation in multiple species, was a crucial starting point for our research. The gene annotation system GENOA is a suite of programs for the spliced alignment of sets of mRNA sequences and ESTs against a whole genome and was used to align human and mouse ESTs and cDNA sequences (described in more detail elsewhere [32]). GENOA detects matches between a repeat-masked cDNA sequence and genomic DNA using BLASTN and maps the original cDNA to the assembled human genome using the spliced alignment algorithm mRNAvsGen. Subsequently, it detects BLASTN matches between a repeat-masked cDNA sequence and EST sequences and maps ESTs to regions with cDNA-aligned genomic DNA using SIM4 [33] to ensure a high quality of annotation. SIM4 aligns ESTs with genomic sequences containing the cognate genes, allowing for introns in the genomic DNA sequence and a relatively small number of sequencing errors.

We obtained chromosome assemblies (hg13) of the human genome from the University of California at Santa Cruz Web server (http://genome.ucsc.edu), transcript data in the form of about 94,000 human cDNA sequences from the combined GenBank files of gpri and gbhtc (release 134), and human ESTs from the database dbEST in repository 032703. Overall, GENOA aligned about 86,000 cDNAs and 890,000 ESTs, which resulted in about 20,800 gene regions within the human genome that exhibited multi-exon structures. The relatively low number of alignments was due to enforcement of stringent alignment criteria. Only ESTs that had at least partial overlap with a cDNA were aligned to the genome, and only those alignments that spanned at least one intron and that met stringent coverage (>90%) and identity levels (>90%) were considered. In the same manner, GENOA was applied to the mouse genome, taking version 3 of the assembly and the same releases of GenBank and dbEST as above. With the same criteria as used for the human data, we successfully aligned about 19,000 cDNAs and 480,000 ESTs, leading to 14,800 gene regions.

For candidate gene regions with alternative exon–intron structures, the spliced alignments were compared for each exon. Annotated 5′ terminal and 3′ terminal exons were separated from internal exons and excluded from further analysis. Internal exons were classified as constitutive, alternative 3′ splice site, alternative 5′ splice site, skipped, overlapping, and containing retained introns. With these alignments and the annotation of orthologs from Ensembl [34], we determined orthologous gene pairs containing conserved AS events. Applying stringent quality filters, we identified a set of 241 skipped exons with corresponding U2-type splice sites in both species that had no other detected AS events involving the skipped exon. This set constituted our test set of known ACEs. Out of the 241 exons, five were masked when applying RepeatMasker (A. Smit and P. Green, unpublished data), showing that some classes of conserved mammalian repeats can lead to conserved alternative exons. Among these five, two were SINEs of the mammalian interspersed repeat (MIR) type, one was an L3/CR1 LINE, one was an ERV class I LTR, and one was a small RNA. A larger number of human skipped exons are derived from primate-specific repetitive elements and therefore not conserved between human and mouse [3].

In the same manner, 5,066 conserved constitutive exons in genes exhibiting AS events elsewhere were identified. From these, we took the 5′ and 3′ splice sites to train the pair splice site output distributions in the model. For a training set for the coding states, orthologous human/mouse coding sequences were extracted from Ensembl, and those coding sequences annotated with start and stop codons in both human and mouse were retained. This set consisted of 5,377 orthologous sequences with known reading frame, totaling 7,140,008 nt in human and 7,005,234 nt in mouse. For the pair states, these sequences were aligned with BLASTN [23]. To prevent predicted exons from including stop codons, stop codons were removed from all coding training sequences, which effectively led to an emission probability of zero for stop codons. Finally, a study on the classification of conserved functional versus nonfunctional sequences provided alignments of 63 conserved functional noncoding regions with a total length of 28,959 nt in human and 28,167 nt in mouse [35].

The analysis of the ENCODE target regions (http://www.ensembl.org/Homo_sapiens/encode.html) was based on the 323 genes located in those regions and annotated by Ensembl as reciprocal best hit orthologs in human and mouse (Ensembl v. 22; June 2004). Our analyses used the Ensembl gene structure annotations of these genes. Orthologous introns were determined by concatenating the flanking 30 nt of both the upstream and downstream exons and blasting these exon junction sequences (EJSs) against all other EJSs from the orthologous gene. The EJS pairs were kept if the alignment extended across the junction and included sequences from both upstream and downstream exons. Identical EJS pairs coming from different transcripts of the same gene were consolidated. Introns were not considered if the intron length was smaller than 40 nt, or if at least one of the flanking exons was shorter than 30 nt. This analysis resulted in 1,823 intron pairs, out of which 1,776 were smaller than 30 kb in both species and subject to our analysis by UNCOVER.

For the analysis of intron retention, we focused on intron pairs in which each sequence was shorter than 1,000 nt, and the difference in length did not exceed 20% of the length of the longer sequence. The retained part together with the flanking exons constitutes one large exon, which is subject to the length restrictions observed for mammalian exons. This is a likely reason why the few known conserved cases of intron retention in mammals all involve relatively short introns of less than 500 nt [19]. In addition to the ENCODE target regions, we determined orthologous introns in the complete human and mouse genomes as annotated by Ensembl. Of the 84,233 orthologous introns, 25,074 satisfied these length restrictions and were analyzed by UNCOVER.

### pHMMs: Structure, implementation, and training.

HMMs provide a probabilistic approach to a large number of problems in computational biology, and have been applied successfully to diverse topics ranging from gene finding to protein domain modeling [20]. A discrete HMM contains a set of states that emit symbols from an alphabet (here, the four nucleotides) according to a probability distribution. The states are connected by transitions, to which probabilities are assigned. A state in such a HMM has an associated probability of observing each residue, and the transitions determine the possible order of the states. A number of dynamic programming algorithms for HMM training and application are well known. The forward algorithm calculates the total probability that a sequence can be generated by a model, and can be applied to classification problems, with several HMMs representing different classes. The Viterbi algorithm yields the parse of a sequence with the highest likelihood, thus assigning the symbols to model states that may represent different functional categories such as exons and introns. pHMMs are extensions of HMMs, originally described to perform local or global alignments of two sequences [20]. In general, the states of the model now contain probability distributions for an alignment of two residues, and by using several different states, a pHMM can be used to model different patterns of conservation. For example, pHMM systems to identify protein-coding genes [36,37] include different states corresponding to pairs of aligned coding and noncoding nucleotides as well as splice sites. The standard HMM algorithms have been generalized and described in more detail for pHMMs [22,37] or, more generally, phylogenetic HMMs [30,38]. When applying the pHMM Viterbi algorithm, we obtain the optimal parse of the alignment into different functional classes along with the alignment, based on the sequence of states used to generate the optimal alignment.

The pHMM data structures and algorithms were implemented in C++ under Linux, with classes for individual model states and the model itself. A command-line interface allows for convenient training of model states, assembling states into a model, and applying the model to align two sequences. States can be either standard single HMM states or pair states and have an associated output distribution that may have *k*-order Markov dependence for a small value of *k*. All single and pair output distributions in the skipped exon model were independently estimated by maximum likelihood using datasets described above. Pseudocounts were added to prevent likelihoods of zero for unseen events, with the exception of the fully conserved U2 splice site dinucleotides and the codon positions (to exclude alignments with stop codons or substitutions of codons of amino acids with very different properties). The Markov order of the output distributions was usually set to one (i.e., the emission probabilities were conditional on the previous nucleotide), with the exception of codon states, where the conditioning was on the previous two nucleotides. As the model topology includes many linearly connected states with a probability of one, only few transition probabilities had to be determined. We derived the gap parameters for functional coding and noncoding sequences from the respective datasets, and manually set the remaining parameters.

With *N* being the number of states in the model, and *L* the length of one input sequence, the run time complexity of the pair Viterbi algorithm to compute the globally best alignment is of order *N* ^2^ *L* ^2^. Thus, many applications of pHMMs, such as comparative gene finding in mammalian genomes, where genes may span across hundreds of kilobases or more, often have to rely on precomputed approximate alignments as input and use the pHMM only to classify and possibly refine the alignment. For the size of most introns, it was practically possible to use the pHMM to compute the optimal global alignment. An optimal pairwise alignment of sequences is usually determined by traceback through the whole dynamic programming matrix. This requires considerable memory resources: the space complexity is *O*(*NL* ^2^), growing quadratically with the size of the input sequences, and for sequences longer than 1,000–2,000 bp each, the forward matrix cannot be fully stored in currently standard main memory any longer. For such sequence pairs, we therefore switched to a divide-and-conquer version of dynamic programming known as the Hirschberg algorithm [39], which reduces the memory requirement to *O*(*NL*) at the cost of doubling the run time: the Viterbi algorithm is started twice in both directions from the beginning and end of the sequences, filling the alignment matrix from both ends up to the center column. During this step, only the currently computed and the previous columns need to be kept, discarding columns computed earlier and thus effectively reducing the memory complexity by one dimension. The sum of the two center columns then contains the score of the optimal alignment, and determines one state transition and pair of symbols within the best alignment. The algorithm is then applied recursively to two subproblems, the alignment from the beginning in the upper left corner to the center split point, and from the center split point to the lower right end of the matrix, reducing the size of the problem by half at each step, which leads to a total doubling in run time.

To increase speed, we used the logarithm of the output and transition probabilities, scaled by −100 and rounded to the nearest integer to limit all operations on probabilities to sums of positive integers. This also ensured that no over- or underflow of numbers occurred. Furthermore, summations in the Viterbi matrix were not taken over all states but only over a list of potential predecessors (those with positive transition probabilities). This list was generated upon loading the model, and provided considerable speedup for sparse transition matrices. We aligned all 241 orthologous intron pairs from the ACE set with the pHMM, ranging in size up to about 90,000 nt each. For practical reasons, we restricted the analysis of the ENCODE region to pairs in which both sequences were smaller than 30,000 nt, setting aside 47 intron pairs longer than that.

The polypyrimidine tract upstream of the 3′ splice site sometimes appears as low-complexity sequence, as do parts of protein-coding regions. We therefore masked only repetitive elements and not low-complexity DNA sequences. In addition, masked sequence was unmasked at both ends by 30 nt, to prevent functional elements from being masked because of neighboring repeats. Repetitive sequences are masked with strings of the letter N, which is treated as a special unalignable character that can only be emitted from single (but not paired) pHMM states. This effectively excludes the possibility that any conserved sequence segments cross masked sequence.

### Experimental RT-PCR validation.

Primer pairs were first designed to the exonic regions flanking the predicted skipped exon (up to 150 nt on each side). We used the Primer3 software (http://fokker.wi.mit.edu/primer3) with the following typical parameter settings: primer length minimum, 18 nt, desired, 21 nt, and maximum, 24 nt; melting temperature minimum, 55 °C, desired, 58 °C, and maximum, 61 °C; product length, 150–250 nt; and prefiltering of potentially mispriming sequences with the provided library of human repeats. A second round of primers included one primer placed within the predicted ACE and one primer in either the up- or downstream exon. Primer sequences were ordered from Invitrogen (Carlsbad, California, United States).

PCR was carried out with the Invitrogen Taq DNA polymerase kit on an ABI GeneAmp 9700 (Applied Biosystems, Foster City, California, United States), with 40 cycles of separation (30 s at 94 °C), annealing (30 s at 55 °C), and extension (45 s at 72 °C). We used BD Biosciences (San Jose, California, United States) Human MTC Panel I normalized cDNA libraries for eight human tissues and HeLa cell line cDNA. For the latter, first strand cDNA synthesis was carried out by incubating total RNA, isolated using TRIzol reagent (Invitrogen), with an oligo(dT) primer at 65 °C for 5 min for denaturing and then placed on ice for annealing. SuperScript III reverse transcriptase (Invitrogen) was used for reverse transcription. We first tested for presence of the predicted alternative spliced exon in brain and liver cDNA, since these tissues were reported to have the highest levels of AS [32]. If not detected or weak, we tested for expression in the six remaining tissues of MTC Panel I (heart, placenta, lung, skeletal muscle, kidney, and pancreas) and in HeLa cells.

PCR products were separated in 2% agarose gels supplemented with ethidium bromide, DNA was visualized under a UV light, and bands were excised and extracted for sequencing on an ABI 3730 DNA Analyzer (Applied Biosystems) using the QIAquick Gel Extraction Kit (Qiagen, Valencia, California, United States) according to the manufacturer's protocol. For weak bands, we performed a second PCR amplification on the extracted bands as described above, to increase the amount of DNA to levels needed for successful sequencing.

## Supporting Information

Dataset S1 Click here for additional data file. Detailed Information on UNCOVER Predictions Verified by RT-PCR or EST Evidence
(5 KB TXT)

Dataset S2 Detailed Information on Predicted Conserved Coding IREs (20 KB DOC)

Figure S1 Expression of Newly Detected Skipped Exons in Different Human and Mouse Tissues (A) Expression of the newly validated exons in human liver tissue and a HeLa cell line. The sample numbering (i–vii) corresponds to the numbers in Figure 2A; the samples, except for sample v, show expression in brain tissue cDNA. Compared with Figure 2A, it can be seen that the inclusion of the skipped exon is tissue specific rather than ubiquitous. Since the PCR product of sample v in Figure 2A was carried out on HeLa cell line cDNA, the reaction shown here (denoted with an asterisk) was carried out on brain tissue cDNA.
(B) PCR results of newly detected isoforms in other human and in mouse tissues (see Figure 2) and validation of the orthologous mouse exons. Roughly half of these were additionally verified by sequencing of the mouse PCR products.
(20 KB DOC)

Protocol S1 Detailed Information on Application and Results of the Ka/Ks Test (29 KB DOC)

### Accession Numbers

The RT-PCR verified sequences described in this paper were deposited in Genbank, under accession numbers DQ102766–DQ102772.

## Abbreviations

ACE: alternative conserved exon
AS: alternative splicing
EJS: exon junction sequence
EST: expressed sequence tag
HMM: hidden Markov model
IRE: intron retention event
NMD: nonsense-mediated decay
pHMM: pair hidden Markov model
