# Supplementary material for: Recognition of Unknown Conserved Alternatively Spliced Exons
Source: PLoS Comput Biol. 2005 Jul 8;1(2):e15. doi: 10.1371/journal.pcbi.0010015 (PMC1185642; doi:10.1371/journal.pcbi.0010015)
Supplement: Dataset S2 — (20 KB DOC) [file pcbi.0010015.sd002.doc]

Supporting dataset S3: Details on predicted conserved coding intron retention events (cf. table 2).

- The EST annotation of Q7M4L6 does correspond to an alternative 3’ss rather than intron retention which is combined with a skipping of the upstream exon (the alternative splice site is too close to the upstream exon to be able to include it).
- The EST overlapping BAT8 also covers only part of the intron and is likely an alternative 3’ss of the downstream exon.
- LACE1 is predicted with slightly higher probability as containing an alternative 3’ss instead of being fully coding throughout the intron, in which case there is no need for to be in frame or a size of a multiple of 3.
- PAX6 is predicted to contain a pax domain spanning across the retained intron.
- The putative retained intron of ENSG00000187999 has 99.6% identity to the intron of HNRPA1, which is annotated as single exon in human but spliced in mouse. Such cases are currently not predicted, as the model assumes a corresponding intron annotation in both species.
