# Supplementary figures and images for: Recognition of Unknown Conserved Alternatively Spliced Exons
Source: PLoS Comput Biol. 2005 Jul 8;1(2):e15. doi: 10.1371/journal.pcbi.0010015 (PMC1185642; doi:10.1371/journal.pcbi.0010015)

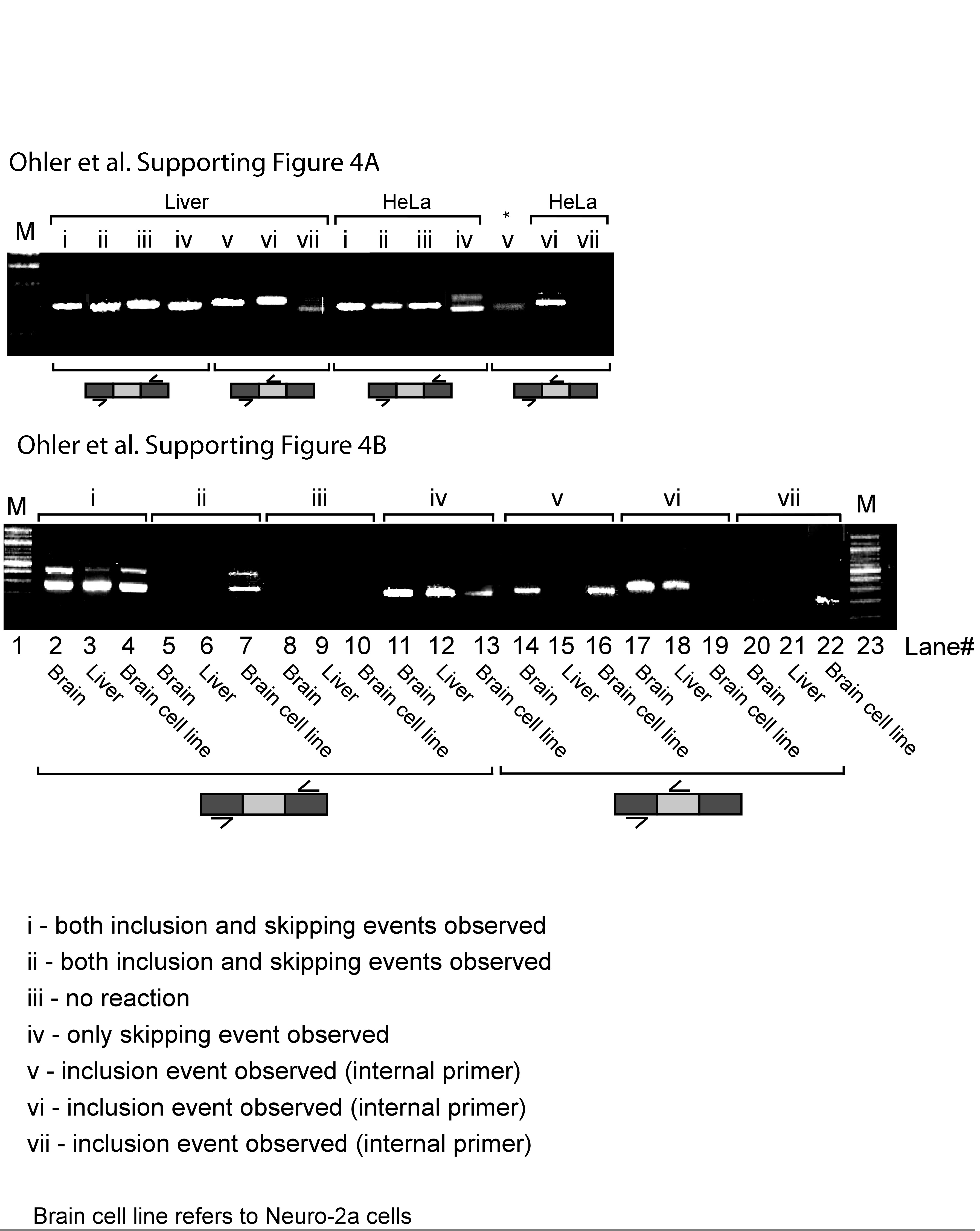

Supplement: Figure S1 — (A) Expression of the newly validated exons in human liver tissue and a HeLa cell line. The sample numbering (i–vii) corresponds to the numbers in Figure 2A; the samples, except for sample v, show expression in brain tissue cDNA. Compared with Figure 2A, it can be seen that the inclusion of the skipped exon is tissue specific rather than ubiquitous. Since the PCR product of sample v in Figure 2A was carried out on HeLa cell line cDNA, the reaction shown here (denoted with an asterisk) was carried out on brain tissue cDNA. (B) PCR results of newly detected isoforms in other human and in mouse tissues (see Figure 2) and validation of the orthologous mouse exons. Roughly half of these were additionally verified by sequencing of the mouse PCR products. (20 KB DOC) [file pcbi.0010015.sg001.doc]
