# Supplementary material for: Recognition of Unknown Conserved Alternatively Spliced Exons
Source: PLoS Comput Biol. 2005 Jul 8;1(2):e15. doi: 10.1371/journal.pcbi.0010015 (PMC1185642; doi:10.1371/journal.pcbi.0010015)
Supplement: Protocol S1 — (29 KB DOC) [file pcbi.0010015.sd003.doc]

# Recognition of unknown conserved alternatively spliced exons in mammalian genomes

Uwe Ohler, Noam Shomron and Christopher B. Burge

# Supporting methods

The Ka/Ks test is an established method to detect adaptive molecular evolution, and has recently been applied to the problem of comparative gene finding. This is an established method to detect adaptive olecular evolution, based on the observation that coding sequences are under evolutionary selection. If Ks denotes the rate of synonymous substitutions per site (here, one codon), and Ka the rate of non-synonymous substitutions, then a ratio of ω=Ka/Ks greater than one points at adaptive evolution where changes are fixed; a ratio smaller than one points at purifying selection; and a value of one corresponds to neutral evolution. As mutation rates are not constant for all genes, Ka and Ks are estimated individually for each exon. A popular approach is implemented in the PAML software package; it uses Maximum Likelihood to estimate the parameters of two nested models (one assuming neutral evolution, and one in which ω is allowed to vary), and a subsequent likelihood ratio test to determine whether the calculated ratio is significantly different from 1^1^. We performed this test using PAML on the 141 of the 241 CSE for which we could infer the reading frame of the exons, which has to be known to estimate the substitution rates per codon. In the application of the Ka/Ks test to gene finding by Nekrutenko^2^, 92% of internal exons passed the test, and several thousand unannotated exons have been predicted using a sliding window along the human genome^3^. However, only 47% of the tested skipped exons passed the test at a P value of 0.05, even despite the unrealistic assumption of knowing the exact exon boundaries. This is apparently due to the smaller size of skipped exons (median 84 nt compared to 123 nt in the set of constitutive exons used by Nekrutenko) and the higher sequence conservation of ACEs compared to constitutive ones, which often makes it impossible to estimate reliable values for Ka and Ks due to the lack of observable sequence variation. The Ka/Ks test therefore has to be considered with extreme caution when applied to detect alternatively spliced exons.

1. Anisimova, M., Bielawski, J.P. & Yang, Z. Accuracy and power of the likelihood ratio test in detecting adaptive molecular evolution. *Mol Biol Evol* **18**, 1585-1592 (2001).

2. Nekrutenko, A., Makova, K.D. & Li, W.H. The K(A)/K(S) ratio test for assessing the protein-coding potential of genomic regions: an empirical and simulation study. *Genome Res* **12**, 198-202 (2002).

3. Nekrutenko, A., Chung, W.Y. & Li, W.H. An evolutionary approach reveals a high protein-coding capacity of the human genome. *Trends Genet* **19**, 306-310 (2003).
